# A General Method for Extracting Individual Coupling Constants from Crowded ^1^H NMR Spectra

**DOI:** 10.1002/anie.201508691

**Published:** 2015-12-04

**Authors:** Davy Sinnaeve, Mohammadali Foroozandeh, Mathias Nilsson, Gareth A. Morris

**Affiliations:** ^1^ School of ChemistryUniversity of ManchesterOxford RoadManchesterM13 9PLUK; ^2^ Department of Organic and Macromolecular ChemistryGhent UniversityKrijgslaan 281 S49000GhentBelgium

**Keywords:** ^1^H-^1^H couplings, configuration determination, conformation analysis, NMR spectroscopy, structure elucidation

## Abstract

Couplings between protons, whether scalar or dipolar, provide a wealth of structural information. Unfortunately, the high number of ^1^H‐^1^H couplings gives rise to complex multiplets and severe overlap in crowded spectra, greatly complicating their measurement. Many different methods exist for disentangling couplings, but none approaches optimum resolution. Here, we present a general new 2D J‐resolved method, PSYCHEDELIC, in which all homonuclear couplings are suppressed in *F*_2_, and only the couplings to chosen spins appear, as simple doublets, in *F*_1_. This approaches the theoretical limit for resolving ^1^H‐^1^H couplings, with close to natural linewidths and with only chemical shifts in *F*_2_. With the same high sensitivity and spectral purity as the parent PSYCHE pure shift experiment, PSYCHEDELIC offers a robust method for chemists seeking to exploit couplings for structural, conformational, or stereochemical analyses.

Homonuclear coupling is a double‐edged sword. On the one hand, it is a well‐established source of valuable information on molecular structure, reflecting torsion or bond angles in the case of scalar couplings, and internuclear vectors in the case of residual dipolar couplings (RDCs).^1^ On the other hand, when, as is almost always the case, multiple couplings are present, the complexity of the resulting multiplets and the narrow range of ^1^H chemical shifts conspire to cause spectral overlap. This complicates spectral analysis and often prevents measurement of individual ^1^H‐^1^H couplings. In recent years, much effort has been invested in generating broadband homonuclear decoupled, or “pure shift”, spectra, collapsing multiplets to singlets and yielding a limiting resolution close to the natural linewidth.^2^ Unfortunately, the gain in resolution comes at the cost of losing direct access to valuable coupling information. Here, we present a method that retains pure shift resolution and high sensitivity, while displaying all couplings to a selected proton or protons as simple doublets in a second dimension. This delivers the maximum information content with minimum complication, making ^1^H‐^1^H coupling measurements straightforward even in challenging molecules.

At first sight, the classic 2DJ spectroscopy (J‐resolved 2D) experiment^3^ ought to provide a good tool for coupling measurements, but it suffers from two severe limitations. First, the presence of a large number of couplings can make analysis of *F*_1_ traces difficult, and limits accuracy. Second, and more seriously, signals are phase modulated and therefore give rise to phasetwist lineshapes.^4^ The classic experiment therefore uses absolute value display, which requires brutal time‐domain weighting functions to be used if lineshapes with acceptable resolution are to be obtained. Unfortunately, this is costly in sensitivity and, crucially, distorts multiplet structure, making the determination of couplings unreliable. The problems of phasetwist lineshapes can be avoided by the use of recent phase‐sensitive 2DJ experiments, although these come at a cost in sensitivity, broadband character, or simplicity of data processing.^5^ The first problem, however, remains.

It is helpful to consider what the characteristics of an ideal 2DJ method for determining coupling constants would be. First, the experiment would be phase‐sensitive, giving absorption mode 2D lineshapes. Second, and importantly, only a subset of couplings of interest would be active, allowing these to be measured without interference from other splittings. Third, it would be broadband, working over the full range of chemical shifts and allowing simultaneous measurement of as many individual couplings as possible. Fourth, and finally, the method would be generally applicable, that is, sensitive, simple to set up, and tolerant of the breakdown of the weak coupling approximation.

The new experiment, dubbed PSYCHEDELIC (Pure Shift Yielded by CHirp Excitation to DELiver Individual Couplings), meets all these requirements. A demonstration is shown in Figure 1 for the steroid 17β‐estradiol. Steroids typically pose challenging cases for the determination of ^1^H‐^1^H coupling constants because of the high incidence of couplings between protons in a narrow chemical shift range. In Figure 1 B,C, only the couplings involving, respectively, proton H9 and proton H14 were selected, using a selective 180° pulse, and appear as simple doublets along *F*_1_ at the chemical shifts of their coupling partners, while all other signals remain as singlets in *F*_1_. Because the experiment has the form of a traditional 2DJ spectrum, the doublets are dispersed at −45° to the principal axes, so applying a conventional 45° tilt (more strictly, a shear) of the spectrum fully removes the active couplings from the *F*_2_ dimension. In this way, full pure shift resolution in the chemical shift dimension (*F*_2_), comparable to that in the parent 1D pure shift PSYCHE experiment, is achieved, reducing the incidence of spectral overlap by almost an order of magnitude compared to the 1D ^1^H spectrum. This is close to ideal for measuring couplings, because even couplings to resonances that differ only slightly in chemical shift can be measured. The coupling constants measured for this region of the 17β‐estradiol spectrum differ significantly in some cases (Supporting Information, Table S2) from the values previously reported,^6^ demonstrating the importance of pure shift resolution for their correct measurement.

**Figure 1:**
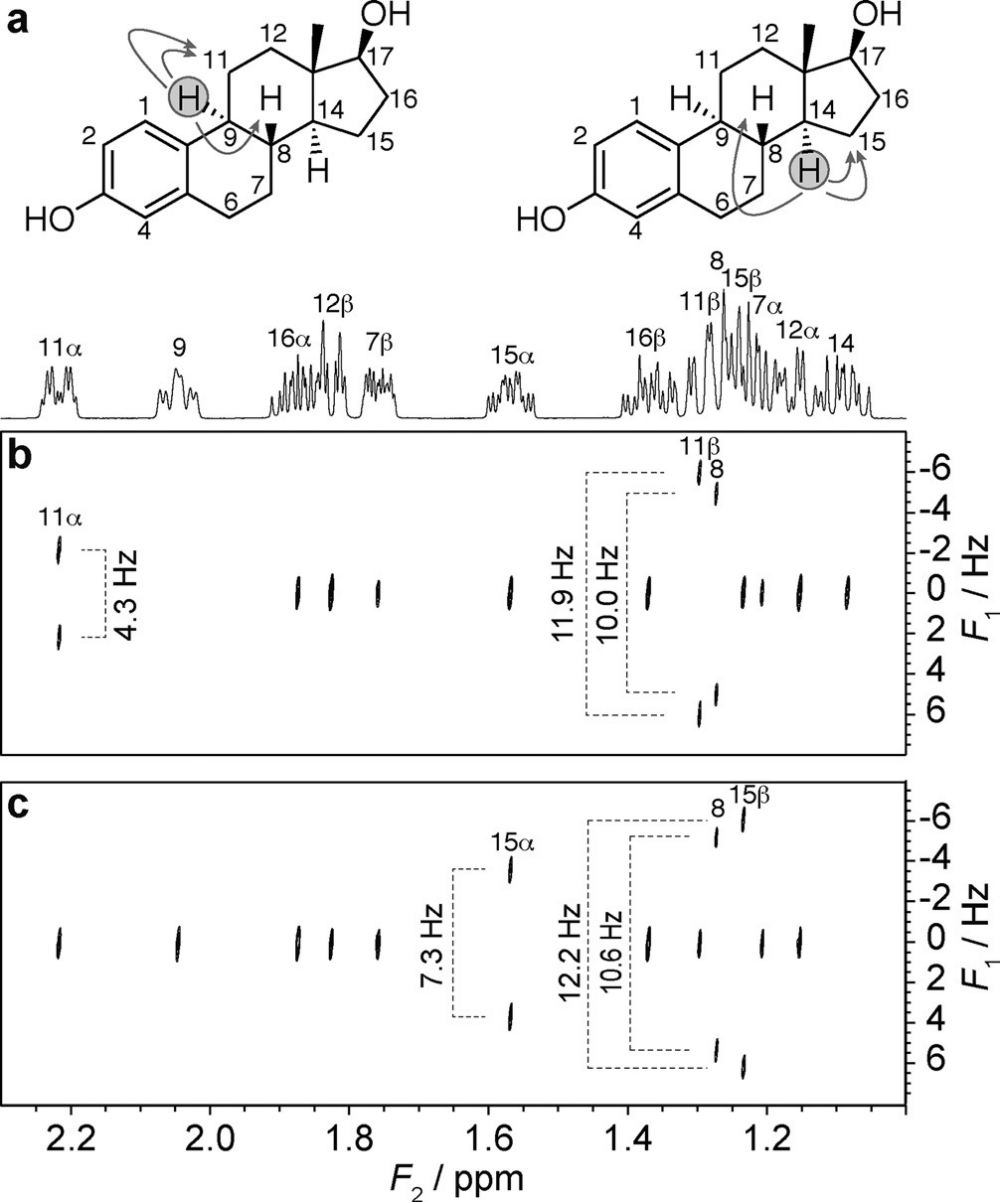
Excerpts from the crowded region of 500 MHz ^1^H NMR spectra of 17β‐estradiol in [D_6_]DMSO, with assignments from reference 6 marked. a) 1D ^1^H spectrum and estradiol structure, indicating the selected couplings involving H9 (left) and H14 (right); b–c) PSYCHEDELIC spectrum (45° tilted) with selective pulses applied to protons H9 (b) and H14 (c).

A complementary demonstration, in which a whole family of spins, rather than a single spin, is selected, is provided in Figure 2, where for the immunosuppressant peptide cyclosporin A all of the spins in the H^α^ region were selected. The resolution offered by PSYCHEDELIC allows, in a single experiment, the measurement of all 18 ^3^ *J* couplings between the H^α^ protons and their coupling partners within both the main and side‐chains.

**Figure 2:**
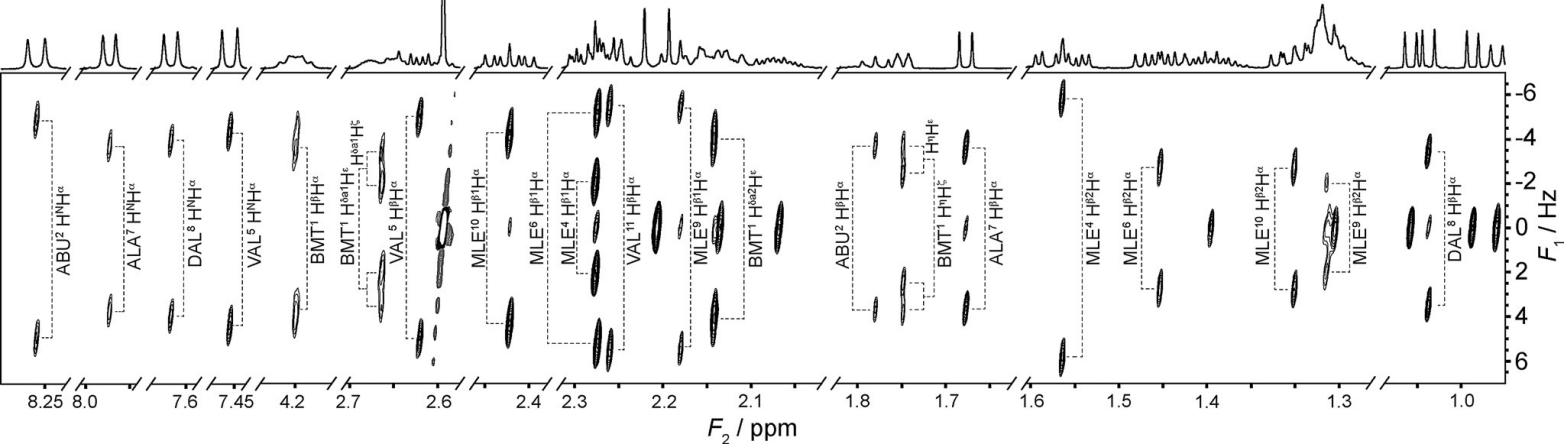
Segments of a 1D and a single PSYCHEDELIC ^1^H spectrum (45° tilted) of cyclosporin A in [D_6_]benzene at 500 MHz, with the selective pulse set to span the H^α^ region. Contours are optimized independently for each segment. Splittings are labeled according to the assignments provided in the Supporting Information. Alkene BMT H^*ϵ*^ and H^ζ^ side chain protons are within the frequency band selected, so spins coupled to both show doublets of doublets.

The way in which selective J‐evolution is achieved during *t*_1_ in the PSYCHEDELIC pulse sequence (Figure 3) is similar to that in the SERF^7^ and G–SERF^8^ methods, but with two crucial differences. First, the PSYCHE2d pulse sequence element is used instead of a band‐selective pulse or a Zangger –Sterk (ZS)^9^ element. PSYCHE allows broadband refocusing of all unselected J‐couplings at high sensitivity, independent of the shift difference between coupled spins, and is thus the best choice for crowded spectra, such as for estradiol (see the Supporting Information). Second, instead of using a *z*‐filter to achieve absorption‐mode lineshapes, as in SERF and G‐SERF, the Pell–Keeler (PK) method5a is used. This combines the results of sequences with normal (N) and reversed (R) evolution in the same manner as classic echo/antiecho processing,^10^ leading to a 2DJ spectrum with double absorption mode lineshapes. A key requirement of the PK method is that the state of the chosen spin during the direct acquisition time *t*_3_ must be the same as that during *t*_1_, for both *N*‐ and *R*‐type acquisition. For conventional 2DJ spectroscopy, in which all couplings are active, this can be achieved using ZS,5a PSYCHE,5f or band‐selective methods.5d In PSYCHEDELIC, only couplings to the chosen spin(s) are active in *t*_1_, so only this state need be preserved (Figure 3). The *N*‐ and R‐type sequences differ only in the location of the *t*_1_ evolution periods. Finally, to achieve suppression of all unselected couplings in *F*_2_, a second evolution period (*t*_2_) is folded around the same PSYCHE element and one of the selective pulses already used for the selective 2DJ evolution. This period samples the evolution of chemical shift and only the selected couplings. For each increment in *t*_2_, a chunk of data of duration 1/SW_2_ is acquired in *t*_3_. These chunks are then combined, just as in other interferogram‐style pure shift experiments,^2^ to give a 2DJ dataset in which the effects of all couplings except that (or those) selected have been suppressed.

**Figure 3:**
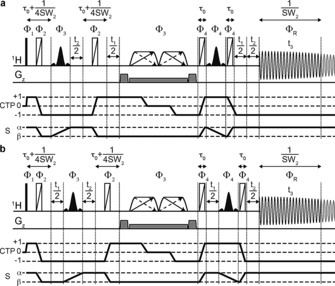
PSYCHEDELIC pulse sequence, a) *N*‐type and b) R‐type. Narrow rectangles indicate 90° RF pulses, and trapezoids with double arrows indicate low‐power chirp pulses of net flip angle *β*, sweeping frequency in opposite directions simultaneously.2d, 5f Wide rectangles with diagonal lines are BIP720 180° pulses.^14^ Black‐filled shaped pulses are selective 180° pulses applied to the selected spin S. Pulsed field gradients are shown on the line G_z_. The highlighted part of the FID, of duration 1/SW_2_, shows the chunk of data acquired for each increment in *t*_2_. The coherence transfer pathway (CTP) selected and the evolution of the state of the selected spin S are shown. The phase cycle is *Φ*_1_=0_4_2_4_; *Φ*_2_=0_8_2_8_; *Φ*_3_=0123; *Φ*_4_=0_16_2_16_; *Φ*_R_=−*Φ*_1_+2 *Φ*_3_.

Two very recent extensions of the SERF and G‐SERF methods have been proposed that also achieve homodecoupling along *F*_2_ using, respectively, the interferogram‐based band selective method (BSD SERF),^11^ and a real‐time ZS method (push‐G‐SERF).^12^ Such approaches can work well in many systems, but fail in the more general and challenging cases in which the chemical shifts of coupled spins are close, such as estradiol (see the Supporting Information). Moreover, the use of seven and six selective pulses, respectively, in the evolution sections of BSD SERF and push‐G‐SERF both imposes a significant further signal loss, and makes these experiments challenging to set up. In contrast, PSYCHEDELIC provides a robust experimental setup, making efficient use of only two simple selective 180° pulses and a single PSYCHE element to achieve multiple goals simultaneously.

There are two special cases where spectral overlap still causes problems in PSYCHEDELIC: the rather rare situation where neither of two coupling partners can be selectively inverted without perturbing a third spin that is also coupled to the detected spin; and the appearance of artifacts when spins are very strongly coupled, a problem common to all 2DJ and pure shift methods. The sensitivity of PSYCHEDELIC is rivaled only by the band‐selective BSD SERF method,^11^ but the latter has greater constraints imposed by spectral overlap, and is limited to observation of a (typically narrow) band of chemical shifts within which there are no mutual couplings.

The pulse sequence of Figure 3 uses interferogram‐style acquisition, with data acquired in pseudo‐3D mode. However, when spectral overlap is not severe, much faster experiments are possible using the same pulse sequence but incrementing only either *t*_1_ or *t*_2_. Incrementing only *t*_1_ provides an absorption‐mode non‐pure shift 2DJ spectrum with selective coupling evolution, similar to G‐SERF but with slightly improved resolution, while incrementing only *t*_2_ results in a 1D pure shift spectrum with only the selected coupling(s) reintroduced, similar to the recent real‐time SERF and QG‐SERF methods^13^ (Supporting Information, Figure S1).

In conclusion, PSYCHEDELIC offers an ideal solution for homonuclear coupling measurements, for the first time making this a straightforward task for very crowded spectra. It gives double absorption‐mode lineshapes and pure shift resolution, resulting in maximum resolving power for couplings. The technique is easily set up, delivers excellent sensitivity and spectral quality, and has very few limitations with respect to spectral overlap. We expect it to be of great value in any application where the accurate measurement of scalar or residual dipolar couplings is required, such as the conformational and configurational analysis of complex organic molecules, including natural products, saccharides, and peptides.

## Supporting information

As a service to our authors and readers, this journal provides supporting information supplied by the authors. Such materials are peer reviewed and may be re‐organized for online delivery, but are not copy‐edited or typeset. Technical support issues arising from supporting information (other than missing files) should be addressed to the authors.

Supplementary

## References

[1a] B. Böttcher , C. M. Thiele in eMagRes, Vol. 1 (Ed.: R. Wasylishen), Wiley, Chichester, 2012, pp. 169–180,

[1b] G. Kummerlöwe , B. Luy , TrAC Trends Anal. Chem. 2009, 28, 483;

[1c] W. A. Thomas , Prog. Nucl. Magn. Reson. Spectrosc. 1997, 30, 183.

[2a] J. A. Aguilar , S. Faulkner , M. Nilsson , G. A. Morris , Angew. Chem. Int. Ed. 2010, 49, 3901; 10.1002/anie.201001107 20401889

[2b] J. A. Aguilar , M. Nilsson , G. A. Morris , Angew. Chem. Int. Ed. 2011, 50, 9716; 10.1002/anie.201103789 21882316

[2c] R. W. Adams in eMagRes, Vol. 3 (Ed.: R. Wasylishen), Wiley, Chichester, 2014, pp. 295–309,

[2d] M. Foroozandeh , R. W. Adams , N. J. Meharry , D. Jeannerat , M. Nilsson , G. A. Morris , Angew. Chem. Int. Ed. 2014, 53, 6990; 10.1002/anie.201404111 PMC4320760 24861024

[2e] K. Zangger , Prog. Nucl. Magn. Reson. Spectrosc. 2015, 86–87, 1. 10.1016/j.pnmrs.2015.02.002 25919196

[3a] W. P. Aue , J. Karhan , R. R. Ernst , J. Chem. Phys. 1976, 64, 4226;

[3b] G. A. Morris in Encyclopedia of Magnetic Resonance, Vol. 9, 2nd ed. (Eds.: R. K. Harris, R. Wasylishen), Wiley, Chichester, 2009, pp. 5205–5216,

[4] G. Bodenhausen , R. Freeman , G. A. Morris , D. L. Turner , J. Magn. Reson. 1978, 31, 75.

[5a] A. J. Pell , J. Keeler , J. Magn. Reson. 2007, 189, 293; 17900950 10.1016/j.jmr.2007.09.002

[5b] S. Simova , H. Sengstschmid , R. Freeman , J. Magn. Reson. 1997, 124, 104;

[5c] B. Luy , J. Magn. Reson. 2009, 201, 18; 19700354 10.1016/j.jmr.2009.07.025

[5d] C. Lendel , P. Damberg , J. Biomol. NMR 2009, 44, 35; 19330299 10.1007/s10858-009-9313-3

[5e] A. Verma , B. Baishya , ChemPhysChem 2015, 16, 2687; 26175136 10.1002/cphc.201500377

[5f] M. Foroozandeh , R. W. Adams , P. Kiraly , M. Nilsson , G. A. Morris , Chem. Commun. 2015, 51, 15410. 10.1039/c5cc06293d 26343867

[6] J. X. Guo , R. I. Duclos , V. K. Vemuri , A. Makriyannis , Tetrahedron Lett. 2010, 51, 3465. 21442021 10.1016/j.tetlet.2010.04.077 PMC3063361

[7a] T. Fäcke , S. Berger , J. Magn. Reson. Ser. A 1995, 113, 114;

[7b] L. Beguin , J. Courtieu , L. Ziani , D. Merlet , Magn. Reson. Chem. 2006, 44, 1096; 16991108 10.1002/mrc.1905

[7c] L. Beguin , N. Giraud , J. M. Ouvrard , J. Courtieu , D. Merlet , J. Magn. Reson. 2009, 199, 41. 19398358 10.1016/j.jmr.2009.03.012

[8] N. Giraud , L. Beguin , J. Courtieu , D. Merlet , Angew. Chem. Int. Ed. 2010, 49, 3481; 10.1002/anie.200907103 20391547

[9] K. Zangger , H. Sterk , J. Magn. Reson. 1997, 124, 486.

[10] J. Keeler , D. Neuhaus , J. Magn. Reson. 1985, 63, 454.

[11] J. E. H. Pucheta , D. Pitoux , C. M. Grison , S. Robin , D. Merlet , D. J. Aitken , N. Giraud , J. Farjon , Chem. Commun. 2015, 51, 7939. 10.1039/c5cc01305d 25865550

[12] D. Pitoux , B. Plainchont , D. Merlet , Z. Y. Hu , D. Bonnaffe , J. Farjon , N. Giraud , Chem. Eur. J. 2015, 21, 9044. 25941095 10.1002/chem.201501182

[13a] N. Gubensäk , W. M. F. Fabian , K. Zangger , Chem. Commun. 2014, 50, 12254; 10.1039/c4cc05892e 25183401

[13b] N. Lokesh , S. R. Chaudhari , N. Suryaprakash , Chem. Commun. 2014, 50, 15597. 10.1039/c4cc06772j 25360453

[14] M. A. Smith , H. Hu , A. J. Shaka , J. Magn. Reson. 2001, 151, 269.

