# Supplementary material for: A General Method for Extracting Individual Coupling Constants from Crowded 1H NMR Spectra
Source: Angew Chem Int Ed Engl. 2015 Dec 4;55(3):1090–3. doi: 10.1002/anie.201508691 (PMC4736434; doi:10.1002/anie.201508691)
Supplement: Supplementary file 1 — Supplementary [file ANIE-55-1090-s001.pdf]

Angewandte Chemie
Eine Zeitschrift der Gesellschaft Deutscher Chemiker

Supporting Information

A General Method for Extracting Individual Coupling Constants from Crowded ^{1}H NMR Spectra

Davy Sinnaeve, Mohammadali Foroozandeh, Mathias Nilsson, and Gareth A. Morris*

anie_201508691_sm_miscellaneous_information.pdf

Supporting information

Table of Contents

Mechanism of the PSYCHEDELIC sequence 2

Experimental Section 5

PSYCHEDELIC experiments 5

Sampling only single data planes with PSYCHEDELIC 7

Comparison with G-SERF and push-G-SERF experiments 9

General considerations 9

G-SERF and push-G-SERF experimental section 12

Signal-to-noise ratio comparisons 13

Comparison with conventional 2DJ spectroscopy 13

Comparison with 1D ^{1}H, G-SERF, and push-G-SERF experiments 13

J-couplings measured for estradiol, revising previously reported data 16

Cyclosporin 18

Pulse sequence 19

Set-up instructions 19

Bruker pulse sequence 21

Mechanism of the PSYCHEDELIC sequence

At the center of the PSYCHEDELIC sequence (Figure 3 main manuscript) is a single-spin inversion (SSI) pulse sequence element, which achieves the inversion of a spin without inverting its homonuclear coupling partners (as opposed to a hard 180° pulse). Here, the most convenient SSI element is the PSYCHE element (Foroozandeh et al., *Angew. Chem. Int. Edit.* **2014**, *53*, 6990), as it achieves single-spin inversion in a broadband fashion (i.e., the whole spectrum can be observed) with good sensitivity and very good tolerance of small chemical shift differences between coupled resonances (i.e., of strong coupling), and can be easily implemented. It works by applying two chirp pulses with low flip angle β, simultaneously sweeping frequency in opposite directions (saltire pulses). This will invert only a small, random fraction of the ^{1}H spins in the ensemble. The coherence selection gradient pulses and phase cycling ensure only detection of these inverted spins. The fraction of spins that is inverted without inversion of their coupling partner, which is what is desired here, amounts to $\sin^2(\beta)$, and determines the sensitivity of the method. For a smaller fraction of spins, amounting to $2\sin^4(\beta/2)$, the coupling partner has also been inverted, leading to artifacts. However, the low flip angle β (typically 15°-20°) can be optimized to keep these artifacts below the noise level, while keeping the sensitivity high for the desired signal. The gradient pulse applied during the two chirp pulses suppresses artifacts arising from coherence transfer due to COSY, zero quantum pathways and strong coupling in a way similar to that proposed previously by the group of Keeler (Pell et al., *Magn. Res. Chem.* **2007**, *45*, 296; Thrippleton et al., *Angew. Chem. Int. Edit.* **2003**, *42*, 3938; Thrippleton et al., *J. Magn. Res.* **2005**, *174*, 97). It should be noted that, although PSYCHE generally provides the best results, other SSI elements can be used, each having advantages in particular cases. A simple band-selective 180° pulse could achieve a much higher sensitivity, but at the cost of broadband character and of many more constraints in terms of spectral overlap. The Zangger-Sterk element (Zangger et al., *J. Magn. Res.*

1997, *124*, 486) has the advantage of being tunable to suit a particular spectrum, but incurs severe sensitivity penalties when coupled partners are close. The BIRD element (Garbow et al., *Chem. Phys. Lett.* **1982**, *93*, 504) can be very useful in dealing with certain cases of strongly coupled spins, but its sensitivity is limited by the natural abundance of ^{13}C and it breaks down when geminal couplings are present.

The SSI element is used for two purposes in PSYCHEDELIC. First, placing the SSI combined with a hard 180° pulse in the middle of an evolution period (t_2) samples chemical shift information in the absence of homonuclear coupling evolution, allowing a pure shift spectrum to be reconstructed using interferogram-style pure shift acquisition (Zangger et al., *Prog. Nucl. Magn. Reson. Spectrosc.* **2015**, *86-87*, 1). However, the additional selective 180° pulses applied to spin S ensure that couplings involving this spin do evolve, and thus appear in the resulting spectrum. Second, an evolution period (t_1) wrapped around the SSI and one of the selective 180° pulses on spin S samples only the evolution of couplings involving spin S, in a similar way to that in the G-SERF experiment. The end result is a 2DJ spectrum with only couplings involving spin S in both F_1 and F_2. The PSYCHEDELIC sequence is arranged in such a way that all other couplings refocus in the middle of the data chunk sampled in t_3, while the selected couplings are refocused at the start of the first data chunk when t_1=0, to avoid J-modulation artifacts.

Finally, to achieve full absorption mode line-shapes, the Pell-Keeler method is used (Pell et al., *J. Magn. Res.* **2007**, *189*, 293), in which a second dataset is generated in which the accumulated precession phase due to the coupling evolution during the evolution period t_1 is reversed in sign relative to that in the first acquisition (Bachmann et al., *J. Magn. Res.* **1977**, *28*, 29; Freeman et al., *J. Magn. Res.* **1979**, *34*, 663). Previous experiments using the Pell-Keeler method applied an SSI element to achieve this (Pell et al., *J. Magn. Res.* **2007**, *189*, 293; Lendel et al., *J. Biomol. NMR* **2009**, *44*, 35; Foroozandeh et al., *Chem. Commun.* **2015**, *51*, 15410). A key realization here is that this

reversed precession only has to be achieved for the selected couplings, so reshuffling the t_1 evolution periods with respect to the selective and hard 180° pulses already present achieves this, without the need to add another SSI element to the sequence.

Experimental Section

PSYCHEDELIC experiments

All spectra were recorded at 298 K on a Bruker Avance II+ 500 MHz spectrometer with a 5 mm BBO probe equipped with a z-gradient coil with a maximum nominal gradient strength of 53 G cm^{-1}. The duration of the ^{1}H 90-degree pulse was 10.4 μs for estradiol (70 mM in DMSO-d_6) and 10.3 μs for cyclosporin (25 mM in benzene-d_6). The spectral widths SW were set to 5 kHz (10 ppm) in the direct (F_3) dimension, 19.531 Hz (=5000/2^8) in the indirect scalar coupling dimension (F_1), and 50 Hz in the interferogram pure shift dimension (F_2). The carrier frequencies were set to 5 ppm for estradiol and 4.5 ppm for cyclosporin.

The chirp pulses used in the PSYCHE pulse element had bandwidths of 10 kHz and durations of 15 ms each, with a maximum RF amplitude of 45 Hz and flip angle of 15°. The gradient pulse during the PSYCHE pulse element was set to 1.5% of the maximum gradient strength. The CTP gradient pulses had a duration of 1 ms and a strength of 20% of the maximum gradient strength. All gradient pulses were followed by a recovery delay of 1 ms. For the PSYCHEDELIC sequence, the disturbance of the lock signal caused by the gradient pulses was found to induce a very small, but noticeable, shift of all cross-peaks (by ~0.1 - 0.3 Hz) along the F_1 and F_2 dimensions, which was eliminated by keeping the lock hold active during signal acquisition (t_3).

For the BIP pulses, BIP720,50,20.1 pulses were used as provided in the Bruker Topspin 3.2 pulse shape library. Their duration was set to 240 μs, with a power level corresponding to a 30 μs 90° pulse and calculated based on the calibrated hard 90° pulse. To avoid phase distortions, these pulses need to be used in pairs.

For the shaped selective pulses, refocusing rsnob 180° pulses were used, with durations set according to the desired spectral bandwidths to be inverted and their frequency offsets set on the resonance(s) corresponding to the selected spin(s). For the experiments on estradiol, with selected spins H9 and H14, durations were 23.32 ms (100 Hz bandwidth) and 53 ms (44 Hz bandwidth) respectively. For the experiments in cyclosporin, the duration was 3.43 ms (680 Hz bandwidth).

The numbers of both t_1 increments and t_2 increments (number of chunks) were set to 32 for the full PSYCHEDELIC experiment, and 64 for the experiments sampling only t_1 or t_2. For the experiments sampling only t_1 and t_3, the total number of time domain points in the direct dimension was set to 32768. The number of transients was 4 for the estradiol sample, resulting in a total experiment time for a single PSYCHEDELIC experiment of about 2 h 25 min. For the cyclosporin A PSYCHEDELIC experiment, 8 transients were used.

Pure shift data processing was performed using the pure shift macro (AU-program) *pshift*, available from the authors' website http://nmr.chemistry.manchester.ac.uk/?q=node/248. All spectra were processed by zero-filling once in each dimension and applying Lorentz-to-Gauss resolution enhancement window functions. The 45° tilting of the spectra was performed by using the 'tilt' command in Topspin.

Sampling only single data planes with PSYCHEDELIC

It is possible to sample only a single plane of the pseudo-3D PSYCHEDELIC experiment, to give either a 1D or a 2D spectrum. Although this leaves coupling and chemical shift information mixed to a certain extent in the same dimension, this can be useful when couplings are to be measured from spectral regions where resolution is less of a concern, as it reduces experiment time.

Sampling only t_1 and t_3 delivers a 2DJ spectrum that provides a similar outcome to the G-SERF experiment (Giraud et al., *Angew. Chem. Int. Edit.* **2010**, *49*, 3481) (Figure S1B). The main advantage is that its limitations in terms of sensitivity and tolerance to strong coupling are determined by the PSYCHE pulse sequence element instead of the Zangger-Sterk. Because it uses the Pell-Keeler method rather than a z-filter (as in G-SERF), it also eliminates the selected couplings from F_2 after 45° tilting, providing a slight resolution advantage compared to G-SERF, which retains the full multiplet in F_2. The passive couplings that remain along F_2 thus limit the resolution in this experiment.

Sampling only t_2 and t_3 (N- and R-type spectra are equivalent when t_1 = 0) delivers a 1D pure shift spectrum (Figure S1C) in which the couplings to the selected spin are not removed from the spectrum, leaving doublets. This is similar to the real-time-G-SERF and QG-SERF experiments, but again with limitations determined by the interferogram PSYCHE pure shift method instead of the real-time Zangger-Sterk method. In this case, the active couplings that remain in the 1D spectrum limit the resolution.

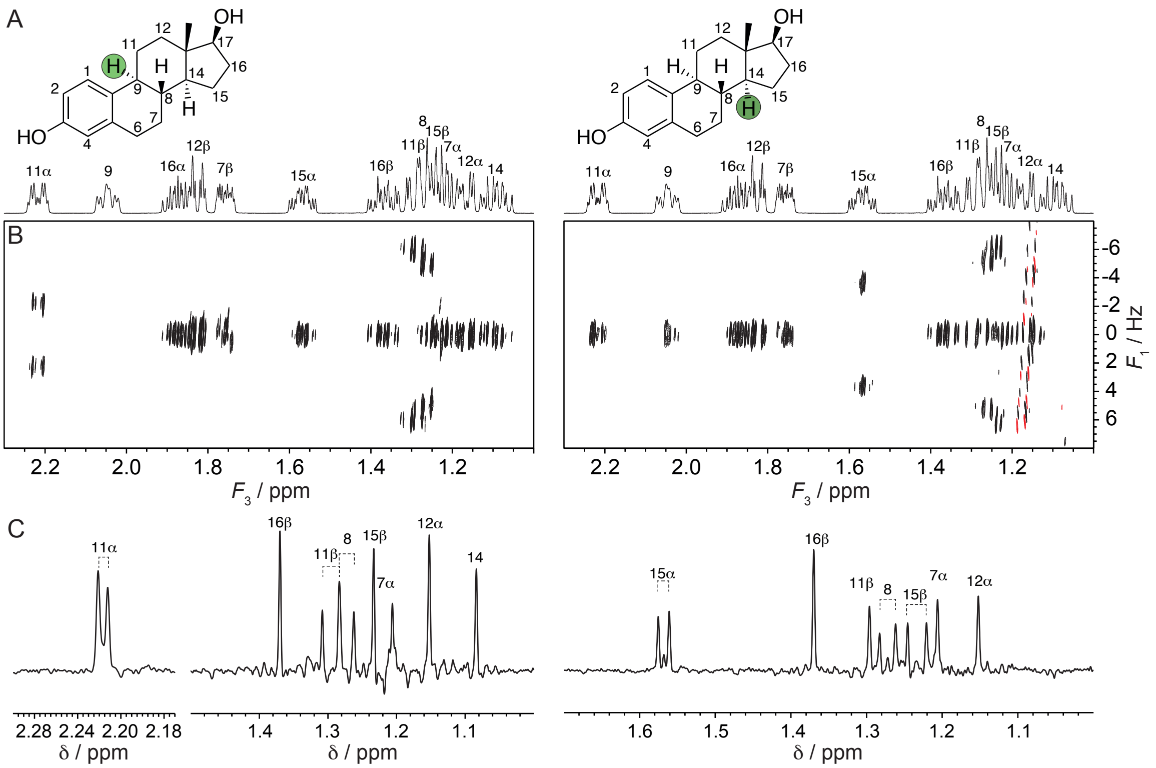

Figure S1. Spectra from PSYCHEDELIC experiments sampling only t_3 and t_1 or t_2. (A) Structure, 1D ^{1}H spectrum and peak annotation of 17β-estradiol. (B) PSYCHEDELIC spectra sampling only t_1 and t_3. (C) PSYCHEDELIC spectra sampling only t_2 and t_3. Left: selective pulse set on H9. Right: selective pulse set on H14.

Comparison with G-SERF and push-G-SERF experiments

General considerations

The G-SERF experiment (Giraud et al., *Angew. Chem. Int. Edit.* **2010**, *49*, 3481) combines the original SERF and Zangger-Sterk (ZS) methods to produce a 2DJ spectrum with absorption mode peaks, and shows only the couplings to a selected spin or spins in F_1. However, because it uses z-filtration to defeat phase modulation, it does not solve the issues of overlap, as it retains the full multiplet structure in F_2 by generating a mirror-image pattern along F_1. This is illustrated in Figure S2B, where G-SERF is applied to 17β-estradiol, with couplings involving H9 (left) and H14 (right) selected to appear in F_1 (compare with Figure 1 in the main paper). Although coupling constants can be extracted from well-resolved multiplets in Figure 1B, analysis of overlapped multiplets (e.g. H8 and H11β) is not at all straightforward. Furthermore, the use of the ZS method, which uses slice-selective pulses, comes at a relatively high sensitivity penalty. This penalty is particularly severe if the weak coupling approximation begins to break down, since this requires the use of highly selective pulses. Since the crowded spectral region of estradiol contains some strongly coupled spins, successful decoupling of these requires a sensitivity loss of at least a factor of several hundred compared to a regular 1D ^{1}H spectrum (see Figure S3).

To solve the problem of overlap in F_2, a recent extension of G-SERF has been proposed that uses a real-time ZS method (push-G-SERF) to give F_2 homodecoupling (Pitoux et al., *Chem.-Eur. J.* **2015**, *21*, 9044). Although such an approach can work well when coupled partners are far apart in the spectrum, it fails for the more general and challenging cases, such as steroids, carbohydrates etc., in which the chemical shifts of coupled spins are close. For push-G-SERF, the real-time ZS acquisition that is used causes severe line-broadening due to the long ZS pulse sequence elements (here at least 60 ms, see Figure S3) needed between data acquisition chunks that typically last only 20-25 ms; the resultant broadening is sufficient to cause overlap again between the spins in the

crowded region (Figure S2C). Interferogram-style acquisition could alleviate this problem, but in the case of estradiol would not solve the inherent sensitivity limitations imposed by using a ZS element.

Another recent experiment delivering couplings at pure shift resolution is BSD SERF (Pucheta et al., *Chem. Commun.* **2015**, *51*, 7939), which concatenates the band selective SERFph experiment (Beguin et al., *J. Magn. Res.* **2009**, *199*, 41) and interferogram-based band-selective pure shift acquisition. Again, although this experiment works well in many circumstances, it would fail in a case like estradiol. First, BSD SERF is not broadband, allowing only a limited chemical shift range to be observed at a time. Second, it only works well when the resonances in the observed band-selected region are each far from all their coupling partners in the spectrum. This condition is not met in the crowded spectral region of estradiol, which contains several mutually coupled spins, and would result in the retention of undesired couplings in both F_1 and F_2 in the resulting spectrum.

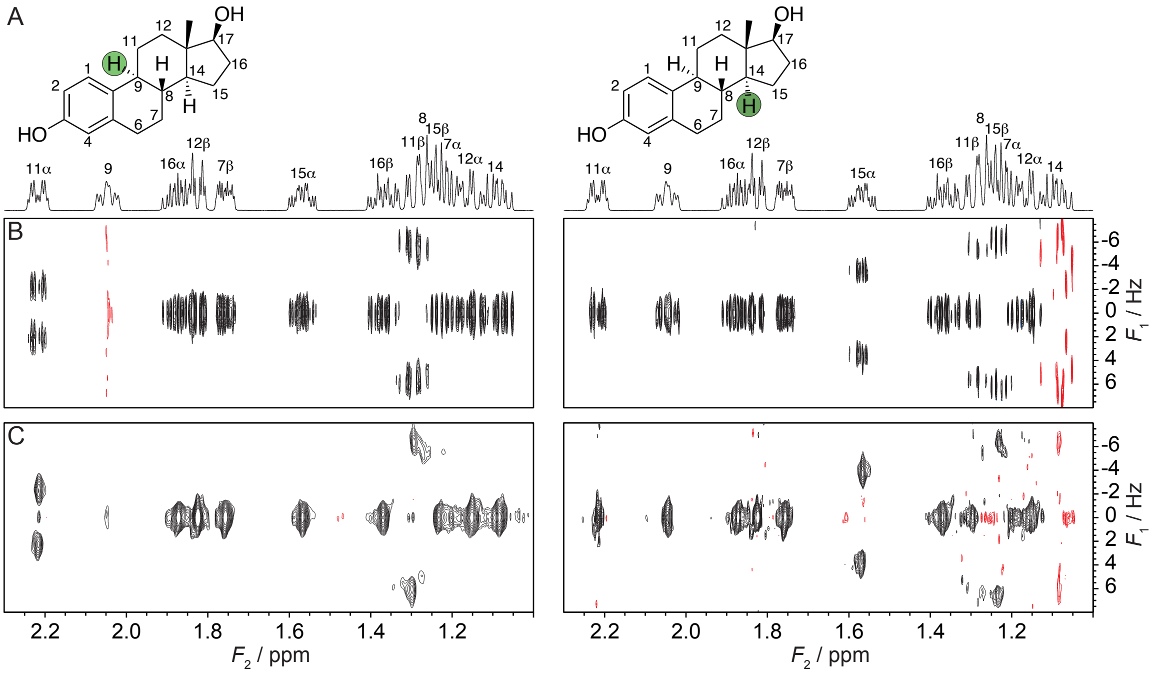

Figure S2. Excerpts from the crowded region of ^{1}H NMR spectra (500 MHz) of 17β-estradiol in DMSO-d_6. The selective pulses are applied to protons H9 (left) and H14 (right). (A) 1D ^{1}H spectrum; (B) G-SERF spectra; (C) push-G-SERF spectra.

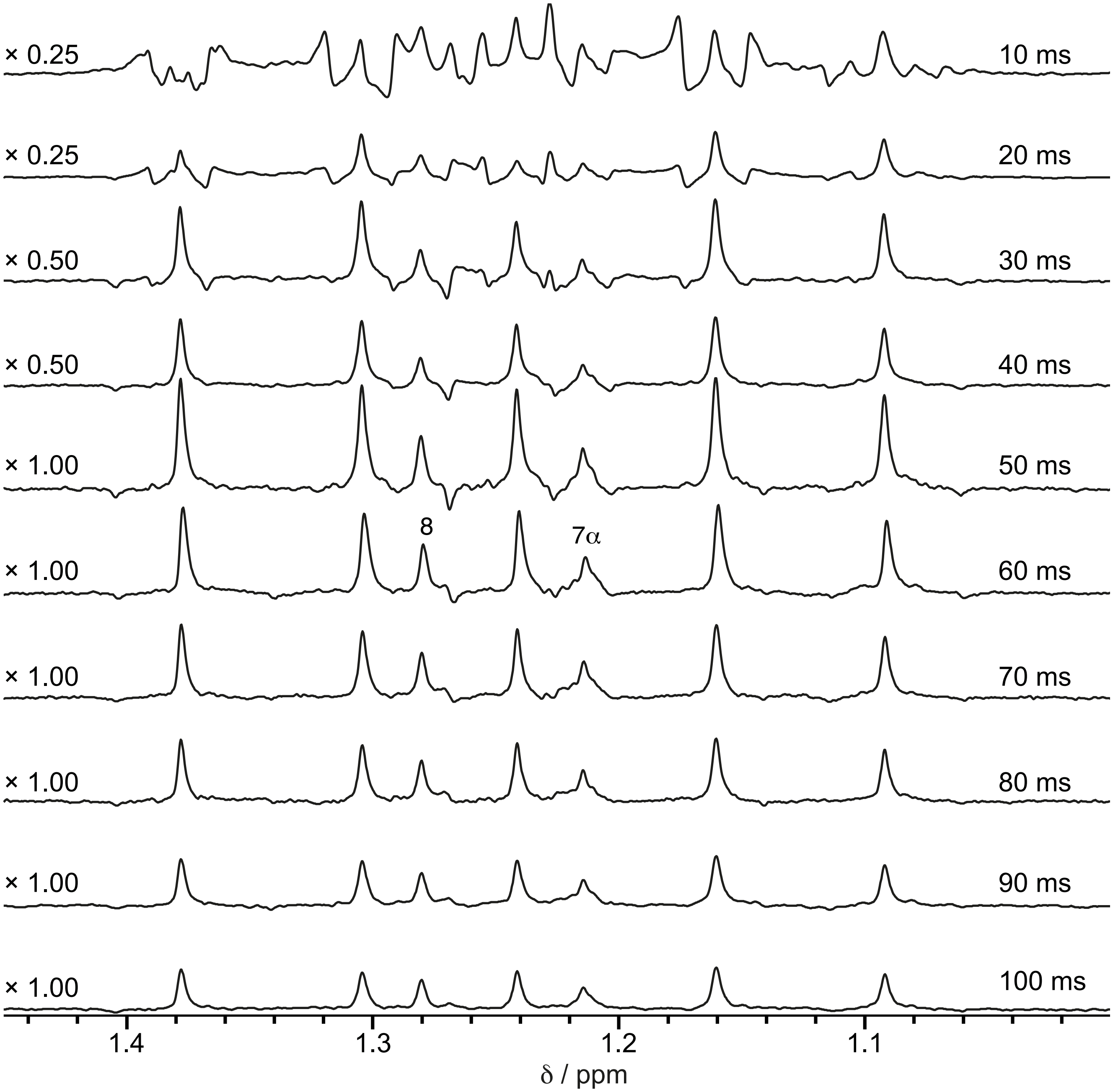

Figure S3. 1D Zangger-Sterk pure shift experiments on 17β-estradiol in DMSO-d_6 at 500 MHz, using the interferogram approach (Aguilar et al., *Angew. Chem. Int. Edit.* **2010**, *49*, 3901; *Angew. Chem.* **2010**, *122*, 3993; pulse sequence available on http://nmr.chemistry.manchester.ac.uk). An rsnob selective pulse was used with a slice selection gradient of 0.8% of the maximum gradient strength. The duration of the Zangger-Sterk element was varied from 10 ms to 100 ms in steps of 10 ms. The relative intensity scale for each spectrum was adjusted as indicated on the left-hand side. Spins 8 and 7α are mutually coupled. The 60 ms rsnob pulse was chosen as a compromise between sensitivity and adequate decoupling of these two spins. Longer ZS elements would also imply further T_2 losses between chunks in the real-time push-G-SERF experiment, and thus further line-broadening.

G-SERF and push-G-SERF experimental section

All spectra were recorded with the same spectrometer and temperature as the PSYCHEDELIC experiments.

For the push-G-SERF experiment, the pulse sequence available in the supporting information of the original reference was used (Pitoux et al., *Chem.-Eur. J.* **2015**, *21*, 9044). For the G-SERF experiment, the same sequence was used, but with the real-time Zangger-Sterk acquisition simply modified to use a standard signal acquisition statement with the go=2 command and an F1EA mc-statement, the latter incrementing a dummy loop parameter in the inner loop.

For G-SERF and push-G-SERF, 60 ms rnsob 180° pulses were used for the Zangger-Sterk (ZS) elements and 41.65 ms esnob 270° pulses for the slice-selective 90° pulses. The duration of the Zangger-Sterk element was 60 ms, which is a compromise between optimal decoupling of the strongly coupled resonances 7α and 8 (ideally requiring ca. 100 ms rsnob pulses) and sensitivity, as illustrated in Figure S3. The slice-selection gradient strength was set to 0.8% of the maximum gradient strength. Selective 180° pulses on the selected spins (H9 and H14) were set up as in the PSYCHEDELIC experiments. Spectral windows in F_2 and F_1 were set to 5000 Hz and 19.531 Hz respectively. For the G-SERF and push-G-SERF experiments, the total number of time domain points in the t_2 dimension was set to 16384 and 4096 respectively. For both experiments, the total number of time-domain points along the t_1 dimension was 32. For push-G-SERF, the number of chunks was 16, implying a chunk duration of 25.6 ms. The CTP gradient pulse duration during the real-time acquisition was 1 ms. The total duration of the J-refocusing element between the chunks was 64.65 ms. For G-SERF, 32 transients were acquired, amounting to a total experiment time of ca. 56 min. For push-G-SERF, 96 transients were acquired, amounting to a total experiment time of ca. 2 h 40 min.

Signal-to-noise ratio comparisons

Comparison with conventional 2DJ spectroscopy

It is not possible to make a direct comparison between the sensitivity of the new experiment and that of a conventional 2DJ experiment (which features the phasetwist lineshape), because the latter requires the use of severe weighting functions and produces quite different lineshapes. However an approximate guide is that the signal loss occasioned by the use of PSYCHE is about an order of magnitude, and that caused by the use of two selective pulses rather less. The signal-to-noise ratio penalty caused by the use of sine bell or pseudo-echo weighting in 2DJ spectroscopy is very variable, but rarely less than a factor of 5 if useful resolution is to be obtained. So the sensitivity penalty of the new method with respect to conventional 2DJ spectroscopy will generally not exceed a factor of 10.

Comparison with 1D ^{1}H, G-SERF, and push-G-SERF experiments

In Figure S4, the signal-to-noise ratios of a 1D ^{1}H spectrum, the first increment (t_1=t_2=0) of a PSYCHEDELIC experiment, and the first increment of a G-SERF experiment are compared for estradiol. The selective pulses, PSYCHE and ZS elements were set up as described in the experimental section. The selective 180° pulses were set on H9. Each experiment was measured using 64 transients, 8 dummy scans, an acquisition time of 2.7s (32768 time-domain points), and a relaxation delay of 1s. Each spectrum was zero-filled once, and no time-domain weighting function was applied. The signal-to-noise ratio was calculated based on the signal region designated “S/N region”.

A similar comparison between the first increments of PSYCHEDELIC and push-G-SERF is shown in Figure S5. The push-G-SERF experiment was measured with the same parameters as the spectra in Figure S4, except for the number of time-domain points (4096) and acquisition time (0.41s).

Including the J-refocusing elements between the chunks (16 chunks), this amounts to a total duration of the whole period needed for signal acquisition of 1.47s. The spectrum was zero-filled to obtain a real spectrum of 32768 points. Gaussian apodization was applied. To allow a fair comparison with the PSYCHEDELIC experiment, for which the same experimental data as in Figure S4 were used, only the first 4096 time domain points of the latter were Fourier transformed in this case, followed by zero-filling to a real spectrum size of 32768 points. The same Gaussian weighting function was applied as for the push-G-SERF spectrum. (The signal-to-noise ratio of a complete PSYCHEDELIC experiment enjoys a further signal-to-noise ratio enhancement because of the collapse of multiplet structure to singlets.)

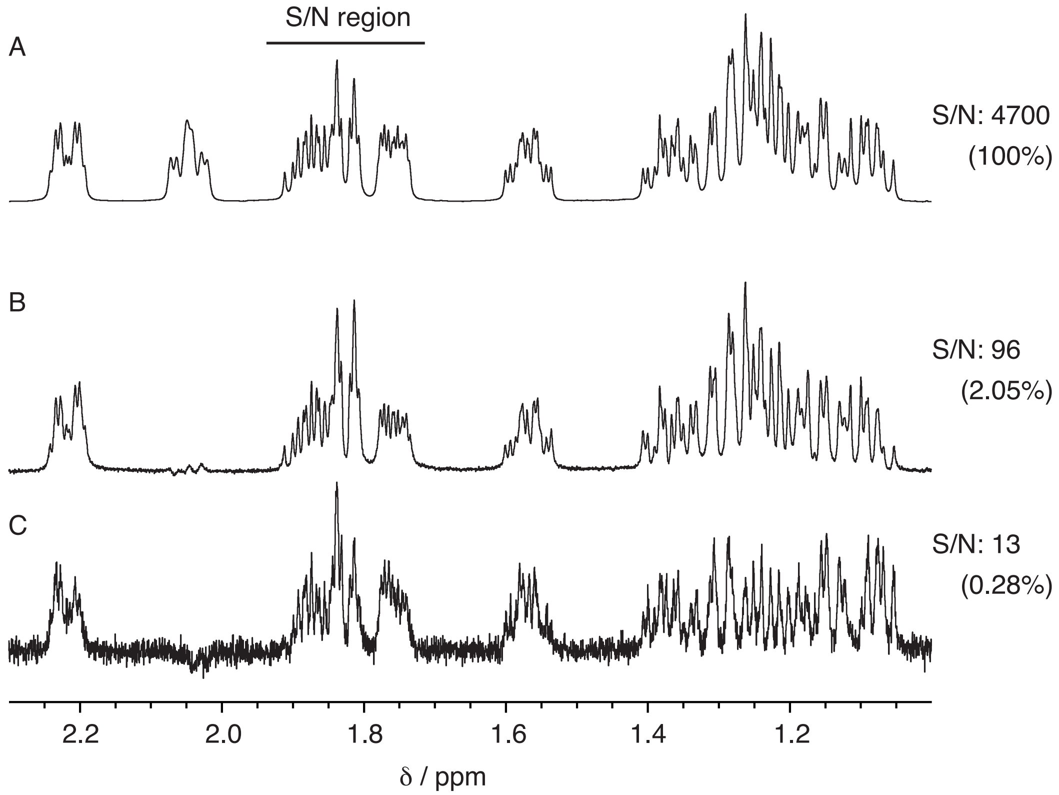

Figure S4. Signal-to-noise ratio comparison between (A) a 1D ^{1}H and the first increments of (B) a PSYCHEDELIC and (C) a G-SERF experiment. The signal-to-noise ratio determined for the region indicated is shown on the right-hand side, as well as the percentage relative to the 1D ^{1}H.

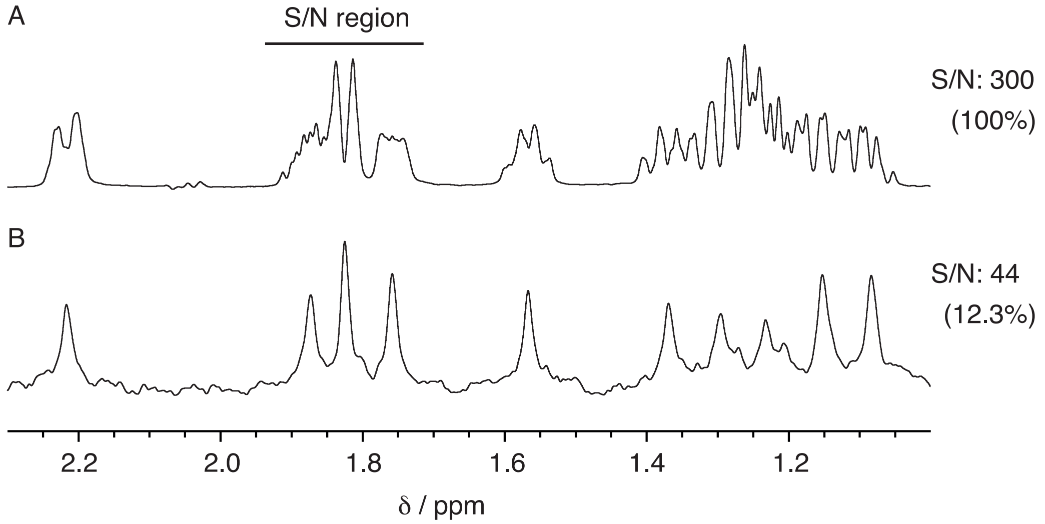

Figure S5. Signal-to-noise ratio comparison between (A) the first increment of a PSYCHEDELIC experiment and (B) a push-G-SERF experiment. The signal-to-noise ratio determined for the region indicated is shown on the right-hand side, as well as the percentage relative to PSYCHEDELIC. Note that push-G-SERF provides a pure shift spectrum in the first increment, whereas in the first increment of PSYCHEDELIC the multiplet structure is still present; the final PSYCHEDELIC spectrum enjoys a further signal-to-noise ratio enhancement because of the collapse of multiplet structure to singlets.

J-couplings measured for estradiol, revising previously reported data

Couplings constants (in Hz) in 17β-estradiol were measured using the PSYCHEDELIC experiments listed in Table S1.

Table S1. PSYCHEDELIC experiments performed to measure ^{1}H-^{1}H couplings in 17β-estradiol

A	Selective pulse set on H16α, H12β and H7β simultaneously
B	Selective pulse set on H14
C	Selective pulse set on H9
D	Selective pulse set on H12α
E	Selective pulse set on H15α
F	Selective pulse set on H2, only sampling t_1 and t_3
G	Selective pulse set on H16β
H	Selective pulse set on H11β, only sampling t_2 and t_3
I	Selective pulse set on part of the H8 multiplet, non-overlapping with the H7α multiplet

In Table S2, the couplings are compared with values reported previously (Guo et al., *Tetrahedron Lett.* **2010**, *51*, 3465.). A few of these couplings (marked in red in Table S2) differ by 1-2 Hz from the previously reported values. All of the discrepancies involve spins in a crowded region of the spectrum, suggesting that the erroneous coupling constants reported previously were a consequence of overlap between multiplets. For example, H8 and H11β, two of the coupling partners of H9, lie very close in the spectrum (0.024 ppm difference), and both the couplings $J_{8,9}$ and $J_{9,11_\beta}$ are found to be wrong. A similar observation applies to H8 and H15β, two couplings partners of H14, (0.039 ppm difference). The PSYCHEDELIC experiment, offering pure shift resolution close to the natural linewidth in F_2 and simple doublets in F_1, avoids such problems.

The protons H6α and H6β could not be disentangled by either PSYCHE or ZS, as these two protons represent a rather severe case of strong coupling (previously reported by Guo et al. using multiplet analysis: $^2J_{6_\alpha,6_\beta}$ = 17.1 Hz and with only a 0.03 ppm chemical shift difference, ca. 15 Hz at 500 MHz).

Couplings to and from these two protons thus could not be measured with PSYCHEDELIC or G-SERF.

Table S2. ^{1}H-^{1}H Couplings measured in 17β-estradiol

Coupling partners	Previously reported [Hz]	PSYCHEDELIC [Hz]	Difference [Hz]	Experiment
1,2	8.5	8.4	0.1	F
1,9	--	1.1		(see Figures S14/S15)
2,4	2.7	2.7	0.0	F
6α,6β	17.1	--		--
6α,7α	6.3	--		--
6α,7β	2.4	--		--
6β,7α	11.6	--		--
6β,7β	6.1	--		--
7α,7β	12.3	12.2	0.1	A
7α,8	12.0	12.2	-0.2	I
7β,8	2.3	2.1	0.2	A
8,9	11.2	10.0	1.2	C
8,14	12.4	10.6	1.8	B
9,11α	4.3	4.3	0.0	C
9,11β	11.2	11.9	-0.7	C
11α,11β	13.5	13.5	0.0	H
11α,12α	4.1	4.1	0.0	D
11α,12β	2.9	2.8	0.1	A
11β,12α	12.8	13.2	-0.4	D
11β,12β	3.8	3.8	0.0	A
12α,12β	12.7	12.5	0.2	A
14,15α	7.4	7.3	0.1	B
14,15β	10.8	12.2	-1.4	B
15α,15b	12.1	12.2	-0.1	E
15α,16α	9.4	9.7	-0.3	A
15α,16β	3.4	3.4	0.0	E
15β,16α	5.8	5.9	-0.1	A
15β,16β	11.5	11.8	-0.3	G
16α,16β	13.4	13.1	0.3	A
16α,17	8.9	9.1	-0.2	A
16β,17	8.4	8.2	0.2	G

For proton labels, see Figure S1. Entries marked in red were found to be significantly different from those reported before.

Cyclosporin

The labels used in Figure 2 in the main paper refer to the cyclosporin A structure below. The selective pulses of the PSYCHEDELIC experiment shown in this Figure spanned all the protons marked with a green circle. All couplings to these protons are measurable from this Figure.

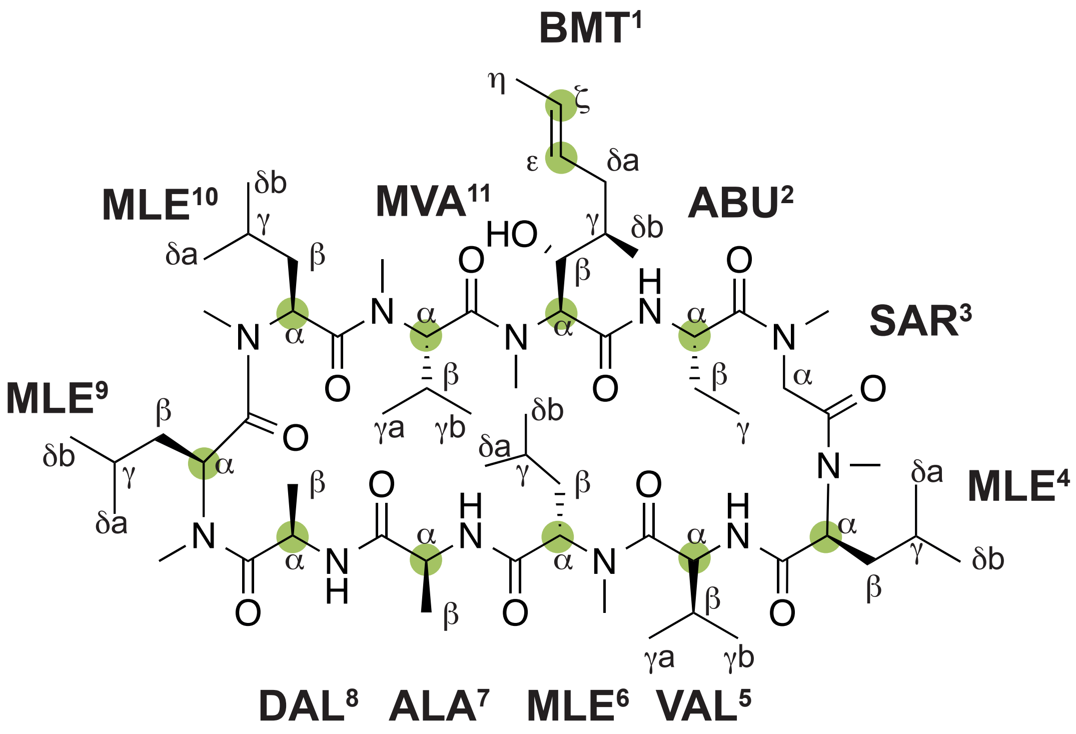

Non-standard amino acid abbreviations: BMT = butenyl-methyl-L-threonine; ABU = alpha-aminobutyric acid; SAR = sarcosin (N-methyl glycine); MLE = N-methyl L-leucine; DAL = D-alanine; MVA = N-methyl L-valine

Pulse sequence

Set-up instructions

The pulse sequence was written and tested in Topspin 3.2. The “F1” column in the acquisition parameters (FnMODE = QF) corresponds to the interferogram t_2 incrementation in Figure 3 of the main paper, while the “F2” column in the acquisition parameters (FnMODE = echo-antiecho) corresponds to interferogram t_1 incrementation. The ratio between the spectral width in Hz (SWH) in the “F3” column and that in the “F2” column is ideally a power of 2 so that the spectra can be tilted (sheared) properly afterwards:

$$\frac{SWH(F3)}{SWH(F2)} = 2^n$$

The ratio between the spectral widths in Hz (SWH) in the “F3” column and the “F1” dimension must be an exact integer N to obtain good quality pure shift spectra:

$$\frac{SWH(F3)}{SWH(F1)} = N$$

Less crucial, but desirable, is that the total number of time domain points in the reconstructed pure shift FID (i.e., after interferogram processing) be a power of 2, to minimize digitization artifacts. To accomplish this, the following relation should be satisfied, with m an integer:

$$\frac{SWH(F3)}{SWH(F1)} \times TD(F1) = N \times TD(F1) = 2^m$$

Since both N and $TD(F1)$ are integers, they both have to be chosen as powers of 2 to achieve the above condition.

The parameter CNST4, the number of extra data points to be discarded from the beginning of each chunk, is typically set as 1 or 2. This reduces artefacts caused by receiver switch-on transients and digital signal processing.

The pulse sequence supports baseopt digitization mode.

The double chirp PSYCHE pulse sequence element is available from the authors' website http://nmr.chemistry.manchester.ac.uk. The flip angle desired for these chirp pulses in degrees is set with CNST20 (see for more details Foroozandeh et al., *Angew. Chem. Int. Edit.* **2014**, *53*, 6990 and Foroozandeh et al., *Chem. Commun. in press*, DOI: 10.1039/C5CC06293D). The power level of the double chirp pulse is calculated automatically within the pulse program from the flip angle, the chirp bandwidth provided in CNST21 (typically 10 kHz), the double chirp element pulse length (p40, typically 30 ms) and the 90° hard pulse calibration p1. The gradient strength during the double chirp is set by gpz12.

The pulse duration of the BIP720 pulses is p10; in our hands, 240 μs provides good results. For this, the pulse shape name (spnam15) should be BIP720,50,20.1. The power level for these pulses is calculated automatically based on the calibrated 90° hard pulse in p1. In principle, the four BIP720 pulses in the pulse program could all be replaced by hard 180° pulses with flanking CTP selection gradient pulses if desired.

For the shaped 180° pulses, the shape name and power level are provided in spnam1 and SPW1, and the shape pulse length in p11. The pulse length and power level required to invert a given bandwidth are easily calculated using the "shape tool" display. The chemical shift of the selected spin (or middle of the region containing the group of selected spins) is provided in CNST1.

Bruker pulse sequence

The PSYCHEDELIC Bruker pulse sequence is provided below. A recent version can be found at http://nmr.chemistry.manchester.ac.uk, as well as pulse sequences for sampling a single plane, and example datasets.

```
;psychedelic3d

; written by Davy Sinnaeve, University of Manchester / Ghent University, 2015
; 2D J-resolved experiment using the Pell-Keeler method
; applying PSYCHE and selective pulses to evolve only selected couplings
; with homodecoupling of the unselected couplings
; run as 3D experiment

; written for Topspin 3.x

; Data can be reconstructed using pshift macro available at
; http://nmr.chemistry.manchester.ac.uk

;
;$CLASS=HighRes
;$DIM=3D
;$TYPE=
;$SUBTYPE=
;$COMMENT=

#include <Avance.incl>
#include <Delay.incl>
#include <Grad.incl>

define delay tauA
define delay tauB
define delay del_corr

"d0=0u"
"d10=0u"
"in0=inf1/2"
"in10=inf2/2"

"p2 = p1*2"

"l0=1" ; switch for Pell-Keeler

;PSYCHE calculations
"cnst50=(cnst20/360)*sqrt((2*cnst21)/(p40/2000000))"
"p30=1000000.0/(cnst50*4)"
"cnst31= (p30/p1) * (p30/p1)"
"spw40=plw1/cnst31"
"p12=p40"

;BIP720 calculations
```

```
"cnst32 = ((p10/8)/p1) * ((p10/8)/p1)"
"spw15 = plw1/cnst32"

;shape pulse offset calculations
"cnst2 = cnst1*bf1"
"spoffs1 = cnst2 - o1"

;pure shift delays
"tauA=in0/2"  ; 1/2SW2
"tauB=(dw*2*cnst4)" ; drop points

; compensation for chemical shift evolution during p1 and group delay
"del_corr = tauA*0.5 + de - p1*2/PI"

"acqt0=0"
baseopt_echo

aqseq 312

1 ze
2 50u
  20u LOCKH_OFF
  d1
  20u pl1:f1
  50u UNBLKGRAD
  p1 ph1
  del_corr pl0:f1    ; tauA*0.5 with corrections
  4u
  (p10:sp15 ph2):f1  ; BIP pulse
  4u
  tauA*0.5

  if "l0 %2 == 0"     ; R-type
     {
     d10            ; Jcoup evolution 2DJ
     }

  4u
  p11:sp1:f1 ph3:r  ; sel. 180 pulse on spin S
  4u

  d0                ; chemical shift evolution

  tauA*0.5
  4u
  (p10:sp15 ph2):f1 ; BIP pulse
  4u
  tauA*0.5

  if "l0 %2 == 1"     ; N-type
     {
     d10            ; Jcoup evolution 2DJ
     }

  tauB              ; extra delay for drop points
  p16:gp1
  d16
  d16
  ( center (p40:sp40 ph3):f1 (p12:gp12) ) ; PSYCHE
```

```
  d16
  p16:gp1
  d16

  4u BLKGRAMP
  (p10:sp15 ph4):f1  ; BIP pulse
  4u

  if "l0 %2 == 0"      ; R-type
     {
     d10             ; Jcoup evolution 2DJ
     }

  4u
  p11:sp1:f1 ph4:r   ; sel. 180 pulse on spin S
  4u

  4u
  (p10:sp15 ph4):f1  ; BIP pulse
  4u pl1:f1

  if "l0 %2 == 1"      ; N-type
     {
     d10             ; Jcoup evolution 2DJ
     }

  d0                 ; chemical shift evolution

  go=2 ph31
  50u mc #0 to 2
    F1QF(caldel(d0,+in0))                  ; pure shift
    F2EA(calclc(l0,+1), caldel(d10,+in10)) ; 2D Jres
  20u LOCKH_OFF
exit

ph1 = {0}*4 {2}*4
ph2 = {0}*8 {2}*8
ph3 = 0 1 2 3
ph4 = {0}*16 {2}*16

ph31= -ph1 + ph3*2

;pl0: 0 W
;pl1 : f1 channel - power level for pulse (default)
;p1 : f1 channel -  high power 90 pulse
;p2 : f1 channel -  high power 180 pulse
;p10: BIP 720 pulse duration
;p11: sel. 180 pulse width for spin S
;p16: CTP gradient pulse duration
;p40: duration of PSYCHE double chirp
;d1 : relaxation delay; 1-5 * T1
;d16: [1ms] delay for homospoil/gradient recovery
;cnst1: Chemical shift of selective pulse (in ppm)
;cnst2: Frequency shift of selective pulse (in Hz)
;cnst4: number of points to drop when collecting FID
;cnst20: desired flip angle for PSYCHE pulse element (degree) (normally 10-25)
;cnst21: bandwidth of each chirp in PSYCHE pulse element (Hz) (normally 10000)
;cnst50: desired PSYCHE RF field for chirp pulse in Hz
;NS: 4 * n, total number of scans: NS * TD0
```

```
;FnMODE: QF in F1
;FnMODE: Echo-Antiecho in F2

;sp1: (spin S) selective pulse power level
;spoffs1: (spin S) selective pulse offset
;spnam1: (spin S) file name for selective pulse
;sp40: (PSYCHE) power level of chirp element (calculated from cnst21)
;spoffs40: (PSYCHE) selective pulse offset (0 Hz)
;spnam40: (PSYCHE) file name for selective pulse
;sp15: BIP pulse power level (calculated from plw1 and p1)
;spnam15: BIP720,50,20.1

; for z-gradients only
;gpz1: 20%
;gpz12: PSYCHE

;use gradient files:
;gpnam1: SMSQ10.100
;gpnam12: RECT.1

;swh in F3 is an integer multiple of swh in F1
;swh in F3 is a power of 2 multiple of swh in F2
```
